# The VISTA Approach in Canine Disimpaction

**DOI:** 10.3390/mps4030057

**Published:** 2021-08-20

**Authors:** Gabriella Galluccio, Alessandra Impellizzeri, Alessandra Pietrantoni, Adriana De Stefano, Gerardo La Monaca, Roberto Pippi

**Affiliations:** Department of Oral and Maxillofacial Sciences, “Sapienza” University of Rome, Via Caserta 6, 00161 Rome, Italy; ale.impellizzeri@gmail.com (A.I.); alessandra.pietrantoni@uniroma1.it (A.P.); aadestefano@hotmail.com (A.D.S.); gerardo.lamonaca@uniroma1.it (G.L.M.); roberto.pippi@uniroma1.it (R.P.)

**Keywords:** impacted canine, miniscrew, VISTA technique, ortho-surgical approach, orthodontics

## Abstract

Canine disimpaction is always a challenging orthodontic treatment overall, even when the impacted permanent canine is in a high position, especially when in tight relation with the upper incisors’ roots. Conventional treatment methods are usually not capable of performing the correct force direction, consisting of the contemporary movement in the distal and vestibular directions of the canine crown, often provoking, as side effects, the presence of decubitus on the mucous of the lips and cheeks or a poor final appearance of the periodontal support of the disimpacted canine. Among the different approaches, the vertical incision subperiosteal tunnel access (VISTA) technique shows good performance with regard to the direction of the forces and the canine’s periodontal conditions when erupted; it is usually realized through an elastic chain connected to a temporary anchorage device (TAD) in the posterior area. In this paper, a different protocol for the VISTA method is also presented, to be resorted to in cases of difficult miniscrew positioning due to the anatomic conditions or stage of dentitions. The new protocol also considers the use of nickel–titanium coil springs in order to avoid the need of frequent reactivation of the device and consequent patient discomfort, highlighting its advantages and indications with respect to the traditional approach.

## 1. Introduction

The orthodontic treatment of an impacted canine is still considered a difficult challenge for clinicians and requires a multidisciplinary therapeutic approach. The therapeutic process often involves the surgical exposure of the impacted tooth, followed by orthodontic traction to guide and align the tooth in the arch.

Surrounding bone loss, root resorption, and gingival recession around treated teeth are some of the most common complications [1,2].

Vestibular or palatal canine impactions recognize different possible etiologies or different relative powers of the same genetic and environmental factors [3,4,5].

A canine position close to the roots of the incisors could lead, during the disimpaction path, to a collision of the tooth crown with them, increasing the risk of root resorption; moreover, the resulting direction of the resultant orthodontic force system could be incorrect and, therefore, unable to carry the canine toward the correct position, sometimes stopping in the orthodontic movement pathway. 

Furthermore, if the position of the canine is in a high position, meaning there is a considerable distance between the tooth crown and the occlusal plane [5], the kind of surgical access and the eruption position could result in a poor final appearance of the periodontal support of the disimpacted canine, meaning a negative outcome for its long-term prognosis [2].

To overcome this problem, various techniques have been proposed to recover an impacted canine toward the desired position, which is capable of exerting a traction in the occlusal and posterior directions, contemporarily adding a vestibular traction. The complexity of the mechanical system has often been the source of side effects, due to the need for high vestibular power arms, which are visible in the painful decubitus on the mucous of the lips and cheeks [6].

Various surgical approaches and orthodontic traction techniques have been described in the literature for maxillary canines impacted in the vestibular area [2].

A recent approach to orthodontic traction of impacted teeth involves the use of the vertical incision subperiosteal tunnel access (VISTA) technique [7,8]. The VISTA technique is a periodontal surgery technique that is generally used for the treatment of single or multiple recessions; it is associated with the insertion of graft material, especially in the anterior sector. It consists of a vertical incision, slightly distant from the area to be treated, followed by a sub-periosteal tunnel by means of a periosteum scraper in which grafting material is to be inserted [9].

The VISTA technique and the use of temporary anchorage devices (TADs) has been suggested for the treatment of impacted teeth including cases of mesio-angled crowns and in tight relation with the root of the lateral incisor. These cases always present a risk of root damage and/or canine eruption in the free gingiva, resulting in a weak appearance of the canine gingiva when erupted [2,7,8,10,11]. 

In the proposed technique, after the surgical exposure of the canine crown through a vertical incision, an orthodontic button is positioned on the crown connected with an elastic chain to a TAD inserted in the zygomatic ridge or in the interradicular space mesial to the first molar.

Several aspects of this technique are sometimes prone to contraindication to the use or to side effects during the traction. 

The placement of the TADs in a position mesial to the first molar is not always feasible when the approach to the canine disimpaction is made during the late mixed dentition phase due to the possible presence of a second premolar bud; likewise, the insertion of the same device in the zygomatic arch is sometimes impossible because of the presence of its lateral extension [12,13,14].

The aim of our work was to present the protocol for the use of this technique in the treatment of impacted teeth but also a modified method for canine disimpaction using the VISTA approach, which is useful for ensuring its application also in the mentioned cases of anatomical hitch, highlighting its advantages and indications compared to the traditional approach, in order to facilitate subsequent canine positioning in its physiological position.

## 2. Experimental Design

The diagnostic procedure, the description of the correct candidate for the clinical approach using the VISTA technique for the impacted canine, and the steps of the clinical procedure are defined. The presentation of two clinical cases in Appendix A help to analyze the phases of the treatment. 

### 2.1. Patient Selection

The candidate subjects should be selected, after the first evaluation, based on the absence of at least one permanent canine after standard eruption time or non-coordinated time with the contralateral canine. Each patient must undergo the following diagnostic procedure:

- Anamnesis;
- Inspection;
- Palpation of the vestibular fornix overlying the lateral incisor to eventually highlight the presence of the bulge of the impacted canine;
- Intra- and extra-oral photos;
- Plaster models;
- X-ray prescription:
  ○ In the first step, a panoramic radiograph and a teleradiography of the skull in latero-lateral projection is required. The observation of the panoramic radiography should show the position of the impacted canine, evaluating the relationships of the canine crown with the roots of the adjacent teeth, the alpha angle, and the distance of the impacted tooth from the occlusal plane. The cephalometric study of the teleradiography should provide information about the eventually present malocclusion and treatment needed;
  ○ A cone beam CT, eventually limited to the sector of the impacted tooth, is mandatory to evaluate the three-dimensional relationships of the teeth with the adjacent structures and all of the conditions eventually limiting the correct insertion of temporary anchoring devices (TADs) such as anatomical variation of the lateral extension of the maxillary sinus, the availability of correct inter-root space, or the presence of the premolars buds in mixed dentition. The use of a tridimensional imaging is justified from the need of an accurate assessment of the position of the impacted canine and of its relation with the adjacent structures, due to the higher spatial resolution obtained with lower radiation dose [15].

### 2.2. Needed Equipment

In cases where TADs are used, a miniscrew of the correct diameter and length is chosen in relation to the selected position, which is usually 1.8 mm in diameter and 8 mm in length for the interradicular position or 2 mm in diameter and 12 mm in length for the infrazygomatic crest. A complete correct surgical kit for miniscrew positioning is necessary; the set is usually composed of 1 screwdriver handle, 1 screwdriver for the contra-angle, 1 manual screwdriver, and 1 drill for site preparation (1.20 mm in diameter, 7 mm in length). In the clinical cases presented, the Firma system (Sweden & Martina, Padua, Italy) was used for the miniscrew selection and surgical kit.

An orthodontic anchorage needs to be bonded on the exposed surface of the impacted canine. Usually, the shape is the orthodontic button, preferably with a curved base, but, depending on the available amount of surface, a small bracket (lower incisor, for instance) or a double cleat can also be chosen. 

**Figure 1:**
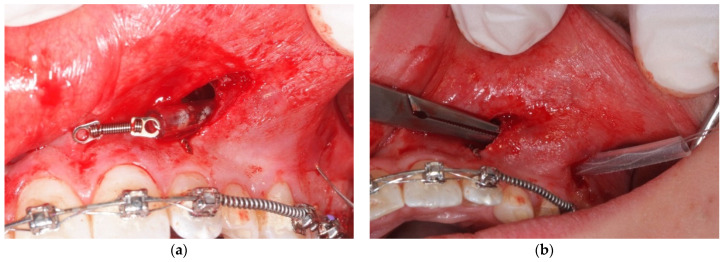
NiTi coil spring 100 gr (**a**) covered with a soft sleeve (**b**) to avoid the insertion of soft tissue in the coils’ loops.

The force is delivered from an elastic chain, as in the original protocol suggested by Zadeh and Chang [7,8], or from an NiTi closed coil spring with 100 gr of force. The advantage of using an NiTi coil is that it avoids the need to reactivate the chain, but in this case, to prevent the possible incorporation of the coil’s loops into the overlying soft tissues, it is better to insert it into a soft sleeve of a correct diameter (Figure 1).

### 2.3. Needed Equipment 2

**Figure 2:**
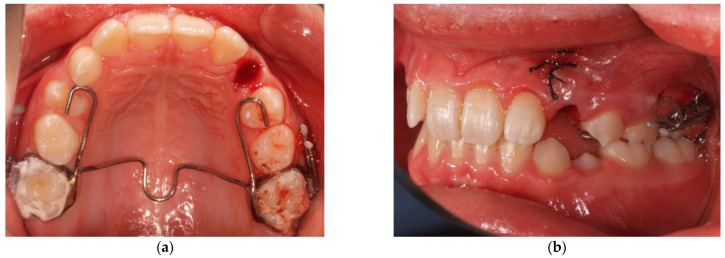
Anchorage through a transpalatal bar (**a**) and a vestibular arm (**b**).

The application of the VISTA technique can be chosen for the recovery of impacted vestibular canines also in cases where the positioning of a miniscrew is impossible due to the above-cited anatomical limitations. The force in these cases can be applied distally on a vestibular arm soldered onto the first molar band of a conventionally designed anchorage device. The anchorage appliance can be a transpalatal bar with stabilizing arms extended to the occlusal surface of the first premolars (Figure 2) or to an expansion fixed device if an upper arch expansion is required for a specific patient such as a quad helix (QH) or a rapid palatal expander (RPE). 

In this eventuality, a preliminary step of choosing the correct band sizes for the first molars, mouth impressions, and the laboratory’s construction of the device is needed. A further appointment is also recommended to check the appliance’s adaptation before the surgical phase.

### 2.4. Needed Equipment 3

#### Surgical Kit 

A few instruments are necessary for surgical canine exposure, sub-periosteal tunnel execution, insertion of the chain or spring inside the tunnel, and the suture: 1 blade-holder with a surgical blade no. 15 c, 1 pair of curved Klemmer forceps, 1 n. 021 multi-blade conical burr, 1 needle holder, 1 pair of scissors, 2 Pritchard-type periosteal elevators, and 1 Vicryk 4-0 suture thread with short tapercut needle. To obtain the hemostasis necessary for the adhesion of the anchoring medium to the canine crown, only a few small pellets of cotton, wet in tranexamic acid or 12% hydrogen peroxide, are necessary.

## 3. Procedure

### 3.1. Orthodontic Surgical Protocol

**Figure 3:**
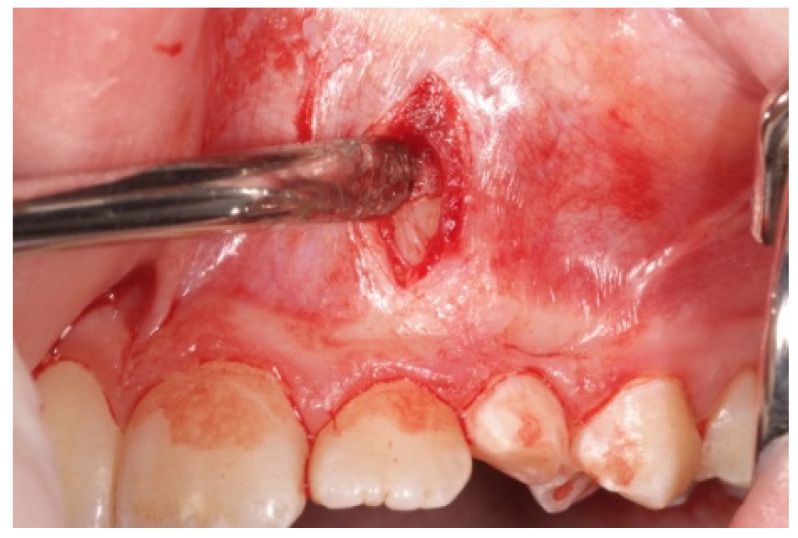
Exposure of the canine crown through a vertical incision.

**Figure 4:**
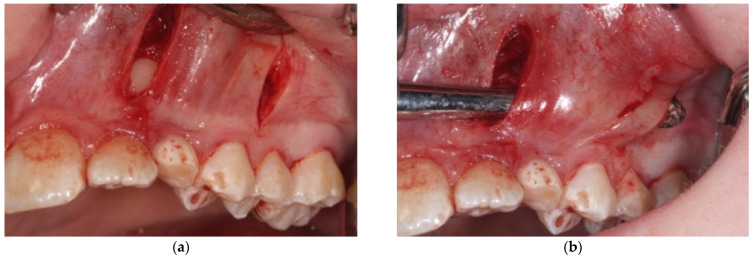
A second vertical incision is made between the first and second premolar (**a**) and connected to the first one through a sub-periosteal tunnel (**b**).

**Figure 5:**
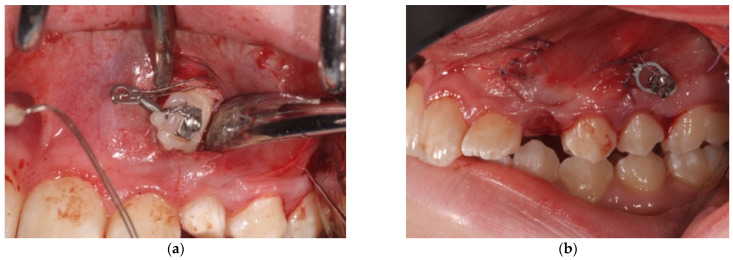
The button is bonded on the canine crown (**a**) and the NiTi coil spring is therefore guided in the subperiosteal tunnel to the posterior anchorage with the aid of a metallic ligature wire (**b**).

After all the diagnostic records have been collected, the procedure is fully explained to the patients and the parents in order to obtain their informed consent. The surgical procedure is a modified version of the orthodontic surgical technique proposed by Chang C. to distalize the crown of impacted canines, moving it toward the correct position, which involves the following operative steps:

1. Local infiltrative anesthesia with vasoconstrictor is performed both in the miniscrew insertion site and at the level of the impacted canine crown;
2. A first vertical incision, at the level of the impacted tooth crown, and its exposure, with a periosteal elevator, are then performed. If a bony cortex is present overlying the crown, a small bony cavity is made using the multi-blade conical burr with a low-speed right-angle handpiece to expose all of the canine’s crown surface (Figure 3). In any case, a thin groove must be present peripherally to the canine’s crown to guarantee a good isolation from bleeding. Moreover, a further ostectomy is performed on the tooth side toward which the orthodontic traction will be performed (traction route);
3. A second vertical incision is made between the first and second premolar, and the sub-periosteal tunnel is created with a periosteal elevator to connect the two incisions from front to back (Figure 4);
4. After a good hemostasis is reached, the button is attached to the buccal canine crown’s surface, following all necessary steps including enamel etching with 37% orthophosphoric acid for 30 s and applying adhesive. Therefore, the button is connected by means of a metal ligature to a 150 gr closed coil spring (NiTi). At the distal end of the NiTi coil, another metal ligature is inserted to facilitate the passage of the traction system under the subperiosteal tunnel (Figure 5);
5. Then, the second metallic ligature is inserted in the subperiosteal tunnel to leak out from the posterior vertical incision. Alternatively, the Klemmer forceps can be inserted through the posterior vertical incision and pushed forward inside the tunnel until its end is visible from the anterior incision, to take the spring end and train it posteriorly until it exits from the rear end of the tunnel;

*Optional step 1 for anchorage provision: miniscrew positioning*

**Figure A1:**
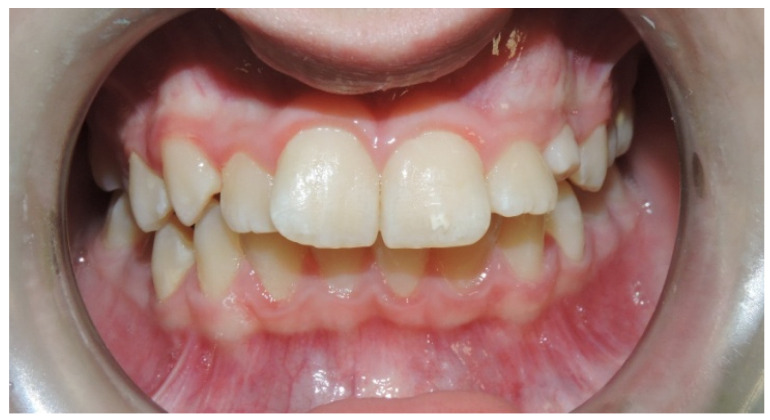
Occlusion of patient B.R.

- The correct site of miniscrew insertion is previously established by means of cone beam CT images, and it is usually planned in the inter-radicular area between the second premolar and the first molar;
- The miniscrew chosen for the present protocol (Firma, Sweden & Martina, Padua, Italy) is 1.8 mm in diameter, 8 mm in length, and it is characterized by a standard head and of a double cross-slot, dimensions 0.022 × 0.022 (Figure A1). It is placed on the impacted canine’s side, at approximately 5 mm from the alveolar ridge, according to the indications of Kocsis [15];
- The miniscrew is inserted, using a special screwer, with a direction forming an angle between 30° and 45° with respect to the occlusal plane;
- After checking the primary stability, the miniscrew is connected to the NiTi spring.

6. Then, the suture of the surgical wounds is performed with an absorbable Vicryl 4-0 thread;
7. The at-home use of 2% chlorhexidine spray is prescribed.

*Optional step 2 for anchorage provision: intraoral appliance*

- After steps 1–5 for canine preparation, the previously prepared intraoral anchorage devices are banded to the first molars with glass–ionomeric cement

The first control is carried out 7 days after the surgical procedure, for wound cleaning and suture removal, and then every month for a routine orthodontic check. After 3 months, the first peri-apical radiography is taken to appreciate the coronal displacement; further radiographic checks with periapical radiography are scheduled every 3 months until the disimpaction is carried out.

Once the impacted canine distalization is obtained, the miniscrew is removed using the special screwdriver, and the closed coil spring is eliminated through a small incision at the crown of the impacted tooth to disconnect it from the orthodontic button.

### 3.2. Time for Completion

The surgical procedure usually takes 45–60 min to perform.

The time needed for the following control visits is usually short, it being only necessary to check the condition of all system components (metallic ligature, miniscrew, or intraoral device), since traction reactivation is not needed due to the shape memory of the NiTi coil spring.

The overall treatment to achieve peri-apical X-ray detectable canine crown distal movement usually takes 3–6 months, while during this period, a monitoring of the eventually present movement of the adjacent teeth can be performed with digital techniques [16]. 

The achievement of the distal movement of the canine crown subsequently allows the uncovering of the canine crown, and a simple extrusion movement toward the occlusal plane can take place.

#### Checklist for the Periodical Control

■ Check the gingival condition around the miniscrew and at the emergence point of the traction metallic ligature. In case of necessity, antiseptic rinses or gel applications are sufficient for inflammation control;
■ Check the miniscrew stability using a tweezer. If necessary, it is possible to screw it again, but a shorter time interval for the next control is appropriate to promptly intercept the possible miniscrew loss;
■ Check the crown position of the contiguous lateral incisor. The displacement of this tooth is related to the pressure exerted on its root by the canine crown during its distal movement. If necessary, a further radiographic control can be performed.

## 4. Expected Results

The VISTA technique, applied to the disimpaction of the vestibular canines, consists of two vertical incisions connected through a sub-periosteal tunnel, under which a traction device is inserted, extended from an orthodontic button or bracket bonded on the canine crown, to an anchorage point, usually a miniscrew, placed distally [9]. 

The aim of the traction to the impacted canine is to let the crown move in the distal direction covered from the gingiva, and it is therefore to be considered as a closed eruption technique.

The principal advantages of the technique are related to the possibility of exerting the orthodontic force in a distal direction also in those cases where there exists a tight connection between the canine crown and the incisors’ roots or in a transposed position between the lateral incisor root and impacted canine root. The orthodontic movement is obtained without the need to involve the incisors in the orthodontic appliance, and the force direction is often capable of inducing an uprighting of the canine’s root axis.

In this study, a variant of the orthodontic surgical technique previously proposed [17,18] was performed: the force necessary to induce the orthodontic movement of the canine was delivered from an NiTi closed coil spring, avoiding the need of the traction’s reactivation typical of elastic chain use, and the subperiosteal tunnel was more extended in length in order to cover the NiTi spring in its entirety. This system allows for the elimination of the mesial small incision intended to permit the passage of the traction outside, as it happens in the use of elastic chain. Moreover, the selection of a more mesial position of the anchorage with a miniscrew located between the second premolar and the first molar or the application on the vestibular surface of the first molar, in cases of conventional intraoral appliances, further increases the ease of the methodology.

The use, as a means of traction, of a NiTi closed spring expresses a continuous and constant force, unlike the elastic chain, which would face the risk of deterioration over a short period of time.

Typical cases of the use of this clinical approach in the treatment of impacted teeth can be framed mainly in these categories:

- ***Vestibular Canine in High Position***: Patients with a canine impacted in the high position—intended as severely distant from the occlusal plane—have an indication to use the modified VISTA technique as the use of other orthodontic traction techniques, such as cantilever or disinclusion arms welded to orthodontic appliances, can often determine the onset of decubitus and periodontal problems, especially in the lower arch. The canines in high inclusion are not covered by adherent gingiva but by alveolar mucosa and can be approached with the excision of a fibro-mucous operculum, with possible removal of a thin layer of bone or with a closed eruption technique with a full-thickness trapezoidal flap [19,20]. Both of these techniques could have periodontal disadvantages of no adherent gingiva on the damage to the marginal periodontium of the adjacent teeth and also related to the initial canine position [20,21,22,23]. In the VISTA protocol technique, no horizontal cuts are provided, avoiding the use of full-thickness flaps that could affect the marginal periodontium of adjacent teeth, since experimental studies on animal models and humans also suggested that the exposure of the alveolar bone that occurs following the execution of a full-thickness flap stimulates the activity of the osteoclasts with a risk of bone resorption [21].
- ***Mesioinclinated Canine in Close Relation to The Root of The Lateral Incisor***: In cases of vestibular canine impaction, a tight relation between the canine crown and the lateral incisor root is often visible. Every so often, moreover, the lateral incisor presents with a horizontal displacement due to the pressure of the erupting mispositioned canine. This condition frequently correlates with an increased risk of root resorption for the lateral and sometimes also for the central incisor. An orthodontic force applied on the canine crown and directed with a distal and occlusal vector could increase the probability of pressure, due to the fact of this latter component, and could therefore increase the risk of root resorption. Conventional treatment should provide a traction arm in a high position and with a horizontal force direction, thus increasing the patient’s discomfort and the likelihood of eruption in free mucosa. Complex and multidisciplinary cases are particularly prone to this difficult operating condition with wide areas of contact between roots and crowns of impacted teeth [24,25]. In any case, a previous evaluation of the disimpaction prognosis is mandatory (angulation, better if the impacted tooth is mesio-angled, α angle, and sector S of localization of the crown according to the classification of Ericson and Kuroll modified by Baccetti [22]).
- ***Adequate/Inadequate Interadicular Space for The Insertion of The Miniscrew***: The positioning of TADs requires a careful choice of the insertion site to select a position useful for the traction of the impacted teeth but respectful of the teeth and of other anatomical structures. The right selection of the patient performed through an accurate study of the radiographic images is essential, preferably on a 3D imaging of the interested arch. A correct inter-radicular space between the first molar and the second premolar is necessary and should be selected adding 1 mm of bone width and 0.5 mm of periodontium width on both sides to the screw diameter measurement. If any adequate inter-radicular space is available, as often occurs in the patient with a mixed dentition, the variant of the technique provides an anchorage to the distalizing force by an intraoral device, usually consisting of a palatal bar with rests on the premolars and a vertical vestibular arm to which the closed coil-spring should be fixed by a metal chain. A similar vestibular arm can in the same way be soldered also on palatal expanders devices eventually needed for the orthodontic therapy.

The VISTA approach provides an approach to traction of the canine with a closed surgical exposure. The choice to pursue the open versus closed surgical exposure is influenced by considerations related to the position and the distance from the occlusal plane of the impacted canine, avoiding results of weak periodontal status of the disimpacted tooth that is primarily related to the kind of surgical exposure [26,27,28,29,30]. Moreover, the closed surgical exposure could sometimes be the only option in cases of multiple inclusion or ectopic position of the canine [5,24,31]. Instead, the second surgical phase of the VISTA technique cannot be intended as an open surgical exposure, because it is dedicated to the traction removal, which is usually performed with the same vertical incision as in the initial phase. The possible need of a vertical traction of the canine, already distalized, is realized with the canine crown positioned in the correct position and at the attached gingiva level. 

An optimal treatment of impacted teeth should be without risk for the adjacent teeth, with optimal periodontal condition and no alveolar bone resorption, achieving the recovery of the canine in the shorter possible treatment duration in order to reduce the overall extension of the orthodontic therapy. Since these treatments also increase the normal duration of orthodontic therapy, the acceleration of the orthodontic movement should be an option. While the use of regional acceleratory phenomenon (RAP) through corticotomies or conductive alveolectomy is questionable in its long-term effectiveness, moreover, linked to the surgical approach used [32,33,34,35], it could be useful to consider the association of this traction methodology and systems with a biostimulating acceleration effect [36,37], thus speeding up the regaining of the correct axial inclination of the impacted canine, which is essential for the recovery. 

**Figure A2:**
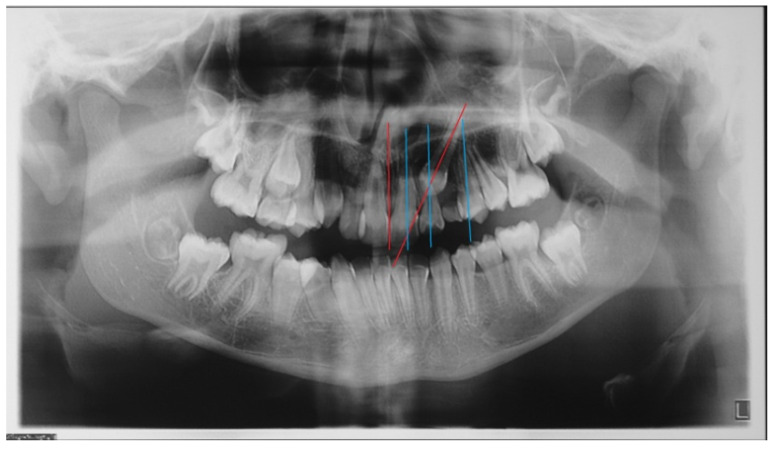
Pretreatment panorex of patient B.R.: vestibular impaction of 2.3, partial superimposition of the canine crown on the lateral incisor root; α angle 26°, sector 3.

**Figure A3:**
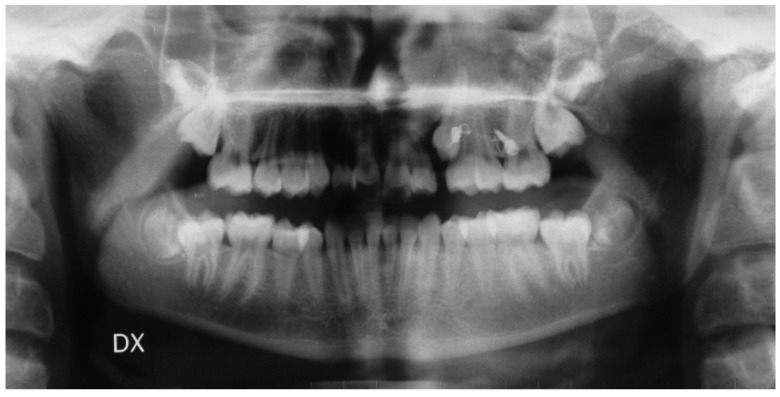
Post-treatment panorex of patient B.R. The distalization of 2.3 was obtained in 6 months.

**Figure A4:**
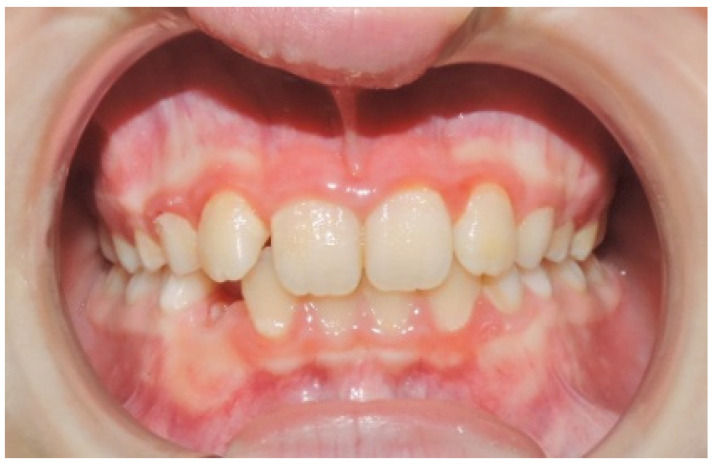
Occlusion of patient L.C.

**Figure A5:**
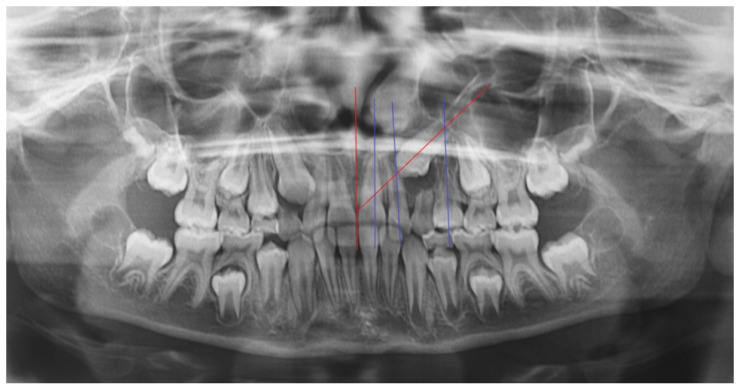
Pretreatment panorex of patient L.C.: high vestibular impaction of 2.3; α angle 48°, α sector 2–3.

**Figure A6:**
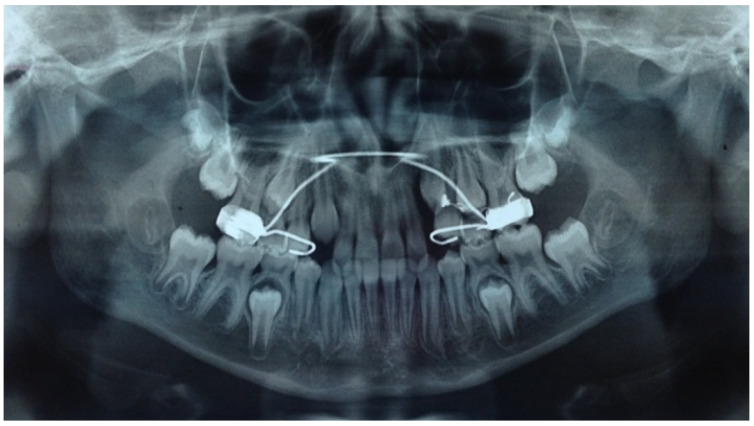
Post-treatment panorex of patient L.C. The distalization was obtained in 4 months.

Two clinical cases illustrating the results of the proposed protocols for the canine disimpaction with the VISTA technique are reported in Appendix A (Figure A1, Figure A2, Figure A3, Figure A4, Figure A5 and Figure A6). 

## 5. Conclusions

The modified VISTA technique may be proposed as an alternative method for the treatment for the treatment of mesio-angulated vestibular canines, both from a biomechanical point of view and from a biological/periodontal point of view.

This work aimed to describe a surgical orthodontic technique for the distalization and disimpaction of mesio-angled vestibular canines, even if in close relationship with the lateral incisor root, and to provide the right clinical recommendations for its correct application. The technique provides advantages but also some disadvantages as summarized in Table 1.

## Figures and Tables

**Table 1:** Summary of advantages and disadvantages of the VISTA approach to canine disinclusion.

Advantages	Disadvantages
**Maximum Anchoring**: The use of a miniscrew, when possible, allows for a maximum anchoring, minimizing side effects. **Compliance**: The use of TADs and closed NiTi springs requests only a minimum compliance from the patient. **Periodontal Advantages**: Distant incision reduces the possibility of traumatizing the gingiva of treated teeth; a critical point is the careful subperiosteal dissection, which reduces gingival margin tension during coronal advancement while maintaining integrity and anatomy of the interdental papillae. The placement of the initial incision and the periosteal tunnel causes almost no visible scarring, helping to maximize the aesthetic outcome in this delicate area. **Comfort for the Patient**: As it is a flapless technique, it is minimally invasive. The patient is also placed in conditions of maintaining proper oral hygiene, given the absence of vestibular orthodontic equipment	**Complications Related to the Use of TADS**: Disadvantages can be related to failure with the loss of the miniscrew, inflammation of the soft tissue, ulceration, root injury, fracture of the miniscrew, pain, and insensitivity. **Second Surgical Phase**: Once the impacted tooth has been distalized, a second surgical phase is required to remove the traction devices (closed NiTi spring and miniscrew).

## Appendix A

**Case #1**

Patient B.R.—Male—13.4 yrs—Vestibular impaction of 2.3.—VISTA Approach with anchorage provided miniscrew.

**Case #2**

Patient L.C.

Female—11.2 yrs—Vestibular impaction of 2.3—VISTA Approach with anchorage provided by an intraoral device.

## References

[1] Manne R., Gandikota C., Juvvadi S.R., Rama H.R., Anche S. (2012). Impacted canines: Etiology, diagnosis, and orthodontic management. J. Pharm. Bioallied Sci..

[2] Becker A., Brin I., Ben-Bassat Y., Zilberman Y., Chaushu S. (2002). Closed-eruption surgical technique for impacted maxillary incisors: A postorthodontic periodontal evaluation. Am. J. Orthod. Dentofac. Orthop..

[3] Yan B., Sun Z., Fields H., Wang L., Luo L. (2013). Etiologic factors for buccal and palatal maxillary canine impaction: A perspective based on cone-beam computed tomography analyses. Am. J. Orthod. Dentofac. Orthop..

[4] Becker A., Chaushu S. (2015). Etiology of maxillary canine impaction: A review. Am. J. Orthod. Dentofac. Orthop..

[5] Galluccio G., Impellizzeri A., De Stefano A.A., Serritella E., Guercio Monaco E. (2020). Multiple Dental Inclusion in Monozygotic Twins with Congenital Visual Impairment. Case Rep. Dent..

[6] Baricevic M., Mravak-Stipetic M., Majstorovic M., Baranovic M., Baricevic D., Loncar B. (2011). Oral mucosal lesions during orthodontic treatment. Int. J. Paediatr.Dent..

[7] Zadeh H.H. (2011). Minimally invasive treatment of maxillary anterior gingival recession defects by vestibular incision subperiosteal tunnel access and platelet-derived growth factor BB. Int. J. Period. Rest. Dent..

[8] Chen C.K., Chang C.H., Roberts W.E. (2014). Class III multiple gingival recession: Vestibular incision subperiosteal tunnel access (VISTA) and platelet-derived growth factor BB. Int. J. Orthod. Implantol..

[9] Bariani R.C., Milani R., Guimaraes Junior C.H., Moura W.S., Ortolani C.L. (2017). Orthodontic Traction of Impacted Upper Canines Using the VISTA Technique. J. Clin. Orthod..

[10] Liou E.J., Huang C. (1998). Rapid canine retraction through distraction of the periodontal ligament. Am. J. Orthod. Dentofac. Orthop..

[11] Impellizzeri A., Palaia G., Horodynski M., Pergolini D., Vernucci R.A., Romeo U., Galluccio G. (2020). Co2 laser for surgical exposure of impacted palatally canines. Dent. Cadmos..

[12] Pellegrino G., Grande F., Ferri A., Pisi P., Gandolfi M.G., Marchetti C. (2020). Three-Dimensional Radiographic Evaluation of the Malar Bone Engagement Available for Ideal Zygomatic Implant Placement. Methods Protoc..

[13] Kuzniak N.B., Fedoniuk L.Y., Pryshlyak A.M., Skyba O.I., Yarema O.M., Dovgalyuk A.I., Penteleichuk N.P., Smiianov V.A. (2020). Morphogenesis of maxillary sinuses in infants, during early and first childhood. Wiad. Lek..

[14] Kocsis A., Seres L. (2011). Orthodontic screw to extrude impacted maxillary canines. J. Orofac. Orthop..

[15] Nardi C., Talamonti C., Pallotta S., Saletti P., Calistri L., Cordopatri C., Colagrande S. (2017). Head and neck effective dose and quantitative assessment of image quality: A study to compare cone beam CT and multislice spiral CT. Dentomaxillofac. Radiol..

[16] Impellizzeri A., Horodynski M., Serritella E., Romeo U., Barbato E., Galluccio G. (2020). Three-dimensional evaluation of dental movement in orthodontics. Dent. Cadmos.

[17] Chang H.N., Su C.W., Hsu Y.L., Roberts E.W. (2011). Soft tissue considerations for the management of impactions. Int. J. Orthod. Implantol..

[18] Chang C.H., Roberts W.E. (2012). Orthodontics, 3Di-Orthoencyclopedia.

[19] Kokich V.G. (2004). Surgical and orthodontic management of impacted maxillary canines. Am. J. Orthod. Dentofac. Orthop..

[20] Chapokas A.R., Almas K., Schincaglia G.P. (2012). The impacted maxillary canine: A proposed classification for surgical exposure. Oral surgery, oral medicine. Oral Pathol. Oral Radiol..

[21] Fickl S., Kebschull M., Schupbach P., Zuhr O., Schlagenhauf U., Hurzeler M.B. (2011). Bone loss after full-thickness and partial-thickness flap elevation. J. Clin. Periodontol..

[22] Baccetti T., Crescini A., Nieri M., Rotundo R., Pini Prato G.P. (2007). Orthodontic treatment of impacted maxillary canines: An appraisal of prognostic factors. Prog. Orthod..

[23] Skidmore K.J., Brook K.J., Thomson W.M., Harding W.J. (2006). Factors influencing treatment time in orthodontic patients. Am. J. Orthod. Dentofac. Orthop..

[24] Impellizzeri A., Midulla G., Romeo U., La Monaca C., Barbato E., Galluccio G. (2018). Delayed Eruption of Permanent Dentition and Maxillary Contraction in Patients with Cleidocranial Dysplasia: Review and Report of a Family. Int. J. Dent..

[25] Impellizzeri A., Giannantoni I., Polimeni A., Barbato E., Galluccio G. (2019). Epidemiological characteristic of Orofacial clefts and its associated congenital anomalies: Retrospective study. BMC Oral Health.

[26] Cassina C., Papageorgiou S.N., Eliades T. (2018). Open versus closed surgical exposure for permanent impacted canines: A systematic review and meta-analyses. Eur. J. Orthod..

[27] El H., Stefanovic N., Palomo J.M., Palomo L. (2020). Strategies for Managing the Risk of Mucogingival Changes during Impacted Maxillary Canine Treatment. Turk. J. Orthod..

[28] Incerti-Parenti S., Checchi V., Ippolito D.R., Gracco A., Alessandri-Bonetti G. (2016). Periodontal status after surgical-orthodontic treatment of labially impacted canines with different surgical techniques: A systematic review. Am. J. Orthod. Dentofac. Orthop..

[29] La Monaca G., Cristalli M.P., Pranno N., Galluccio G., Annibali S., Pippi R. (2019). First and second permanent molars with failed or delayed eruption: Clinical and statistical analyses. Am. J. Orthod. Dentofac. Orthop..

[30] Calasso S., Cassetta M., Galluccio G., Barbato E. (2008). Impacted lower second molars. Dent. Cadmos.

[31] Cavuoti S., Matarese G., Isolac G., Abdolreza J., Femiano F., Perillo L. (2016). Combined orthodontic-surgical management of a transmigrated mandibular canine. Angle Orthod..

[32] Ren A., Lv T., Kang N., Zhao B., Chen Y., Bai D. (2007). Rapid orthodontic tooth movement aided by alveolar surgery in beagles. Am. J. Orthod. Dentofac. Orthop..

[33] Fu T., Liu S., Zhao H., Cao M., Zhang R. (2019). Effectiveness and Safety of Minimally Invasive Orthodontic Tooth Movement Acceleration: A Systematic Review and Meta-analysis. J. Dent. Res..

[34] Darwiche F., Khodari E., Aljehani D., Gujar A.N., Baeshen H.A. (2020). Comparison of Effectiveness of Corticotomy-assisted Accelerated Orthodontic Treatment and Conventional Orthodontic Treatment: A Systematic Review. J. Contemp. Dent. Pract..

[35] Kamal A.T., Malik D.E.S., Fida M., Sukhia R.H. (2019). Does periodontally accelerated osteogenic orthodontics improve orthodontic treatment outcome? A systematic review and meta-analysis. Int. Orthod..

[36] Jedliński M., Romeo U., Del Vecchio A., Palaia G., Galluccio G. (2020). Comparison of the effects of photobiomodulation with different lasers on orthodontic movement and reduction of the treatment time with fixed appliances in novel scientific reports: A systematic review with meta-analysis(Review). Photobiomodulation Photomed. Laser Surg..

[37] Impellizzeri A., Horodynski M., Fusco R., Palaia G., Polimeni A., Romeo U., Barbato E., Galluccio G. (2020). Photobiomodulation therapy on orthodontic movement: Analysis of preliminary studies with a new protocol. Int. J. Environ. Res. Public Health.

